# Heterologous immunization with adenovirus vectored and inactivated vaccines effectively protects against SARS-CoV-2 variants in mice and macaques

**DOI:** 10.3389/fimmu.2022.949248

**Published:** 2022-08-17

**Authors:** Qian He, Qunying Mao, Jialu Zhang, Fan Gao, Yu Bai, Bopei Cui, Jianyang Liu, Chaoqiang An, Qian Wang, Xujia Yan, Jinghuan Yang, Lifang Song, Ziyang Song, Dong Liu, Yadi Yuan, Jing Sun, Jincun Zhao, Lianlian Bian, Xing Wu, Weijin Huang, Changgui Li, Junzhi Wang, Zhenglun Liang, Miao Xu

**Affiliations:** ^1^ Division of Hepatitis and Enterovirus Vaccines, Institute of Biological Products, National Institutes for Food and Drug Control, Beijing, China; ^2^ Guangzhou Laboratory, Guangzhou, China

**Keywords:** COVID-19 vaccine, heterologous immunization, protective effectiveness, animal model, SARS-CoV-2 variants

## Abstract

To cope with the decline in COVID-19 vaccine-induced immunity caused by emerging SARS-CoV-2 variants, a heterologous immunization regimen using chimpanzee adenovirus vectored vaccine expressing SARS-CoV-2 spike (ChAd-S) and an inactivated vaccine (IV) was tested in mice and non-human primates (NHPs). Heterologous regimen successfully enhanced or at least maintained antibody and T cell responses and effectively protected against SARS-CoV-2 variants in mice and NHPs. An additional heterologous booster in mice further improved and prolonged the spike-specific antibody response and conferred effective neutralizing activity against the Omicron variant. Interestingly, priming with ChAd-S and boosting with IV reduced the lung injury risk caused by T cell over activation in NHPs compared to homologous ChAd-S regimen, meanwhile maintained the flexibility of antibody regulation system to react to virus invasion by upregulating or preserving antibody levels. This study demonstrated the satisfactory compatibility of ChAd-S and IV in prime-boost vaccination in animal models.

## Introduction

In response to the COVID epidemic sweeping the world, a variety of vaccines including mRNA vaccines, inactivated vaccines, adenovirus vectored vaccines, and recombinant protein subunit vaccines have reported satisfactory protection efficacy (>50%) against prototype SARS-CoV-2 and have been approved for marketing in the shortest time possible (1, 2). As of February 21, 2022, over 10 billion doses of various COVID-19 vaccines had been administered according to World Health Organization (WHO) statistics (3). Nonetheless, it’s rationale to further investigate boosting strategies to improve population immunity due to the natural decline in antibody levels after vaccination (4–6) and the decreased antibody neutralization activity caused by the prevalence of variant strains (6–10). Therefore, research on heterologous prime-boost vaccination using different vaccine technology platforms has attracted significant international interest.

Heterologous prime-boost regimens (11, 12) have been tested in many studies for developing vaccines against AIDS and influenza (13, 14) and proved to be more effective than homologous prime-boost regimens containing the same vaccine formulations. Heterologous prime-boost immunization includes various strategies. Different strain expression vectors of the same species were used in the immunization procedure to reduce the anti-vector immune response. The approved adenovirus vectored vaccine Sputnic V is a heterologous combined vaccine of rAd26 and rAd5 and exhibits superiority compared to single rAd26 or rAd5 vaccines in phase 1/2 clinical trial (15). In a more complicated situation, a heterologous booster vaccine could have completely different formulations or different expression and presentation systems. Vaccine platforms, such as viral vectored vaccines and DNA vaccines, are characterized by a robust T cell response benefiting from increased MHC-I presentation. However, exogenous antigens, such as inactivated vaccine and recombinant protein subunits, induce a dominant antibody response if adjuvanted by the classic aluminum adjuvant. The novel mRNA vaccines seem to be effective for the aforementioned immune arms, according to currently reported data (16, 17). Prime-boost regimens with endogenous and exogenous vaccines might elicit immune responses in multiple arms, including cellular and humoral responses. Therefore, endogenous and exogenous vaccines are often combined to strengthen and broaden the immune potency of preventive vaccines (18–20).

Our previous work on COVID-19 vaccination strategies illustrated the superiority of the heterologous prime-boost regimen with Ad5 vectored vaccine and inactivated or protein subunit vaccine in inducing protective immune responses in mice (21, 22), which has been further confirmed in a phase 4 clinical trial (23). The chimpanzee adenovirus vector is currently one of the most studied adenovirus vectors in COVID-19 vaccine research and development (1), among which ChAdOx1-nCoV had been approved in WHO emergency use list and administered to a large population, together with two mRNA vaccines and three inactivated vaccines (24). Clinical trials have reported that heterologous prime-boost immunization with ChAdOx1-nCoV and the mRNA vaccine BNT162b2 could achieve quite high level of neutralizing antibody and Th1 biased T cell responses (25–27). Currently, it is necessary to explore the compatibility and immune effects of chimpanzee adenovirus vectored vaccines and inactivated vaccines to support the formulation of global heterologous prime-boost immunization policies. In this study, we evaluated the immunogenicity and protective effect of the heterologous prime-boost regimen with a chimpanzee adenovirus vectored vaccine ChAd-S and an inactivated vaccine IV in mouse and rhesus macaque models and provided supporting data on the compatibility of the two types of vaccines.

## Materials and methods

### Animals and vaccines

The specific pathogen-free female BALB/c mice (6–8-week-old) used in the study were provided by and maintained at the Chinese National Institutes of Food and Drug Control. Female Chinese rhesus macaques (2–3-year-old) were provided by and housed at the Kunming National High-level Biosafety Primate Research Center, China. All experiments with live SARS-CoV-2 in NHPs were performed at the ABSL-4 facility. Inactivated vaccine IV used in this study was an inactivated ancestral SARS-CoV-2 strain isolated at the beginning of 2020. The ChAd-S vaccine was a non-replicable ChAd vectored vaccine expressing full length of spike protein targeting ancestral SARS-CoV-2. The dosage was 1×10^10^ vp for ChAd-S and 1μg for IV in mice; 0.5×10^11^ vp for ChAd-S and 5μg for IV in NHP.

### Enzyme-linked immunosorbent assay

The levels of serum antibodies directed against the SARS-CoV-2 spike protein were assessed using ELISA, as we previously described (21). Ninety six-well EIA/RIA plates were coated overnight with 1 µg/mL recombinant S protein at 4°C. After removal of the unbound spike protein and blocking of the plate with 10% fetal bovine serum in 0.5% PBST, 10-fold serially diluted test samples were added to the wells. The bound antibodies were subsequently detected after incubation with 1:5000 diluted goat anti-mouse IgG (HRP-labeled) (China ZSGB-BIO, cat#ZB2305), followed by development with substrate (China Beijing Wantai BioPharm, cat#N20200722) at 450 and 630 nm. The endpoint of serum antibody titers was determined by the reciprocal of the highest dilution, which was 2.1-fold higher than the optical absorbance of the negative control.

### Serum neutralization assay

The levels of serum NAbs against SARS-CoV-2 were measured using live and pseudo-SARS-CoV-2 viruses, and the results were expressed as GMTs. NAbs against live SARS-CoV-2 (Prototype, Alpha, Beta, Delta, Omicron variants isolated in China) were quantified using a microcytopathogenic effect assay at a minimum 8-fold dilution. The neutralization capacity against a pseudovirus (Wuhan-Hu-1, GenBank: MN908947, optimized for human cell expression) was determined as previously described (28).

### IFN-γ ELISpot assay

The IFN-γ ELISPot assay was conducted as previously described. Freshly isolated splenocytes were stimulated with a pool of peptides spanning the SARS-CoV-2 S protein for 20 h at a density of 2×10^5^ cells per well. The concentration of each peptide was 5 μg/ml. The peptide pool was generated as follows: a panel of consecutive 15-mer peptides with nine overlapping amino acids was synthesized to encompass the entire S protein and mixed as one peptide pool. After stimulation, the plates were incubated with IFN-γ-detecting antibodies. Spots representing IFN-γ-producing cells were counted using an ImmunoSpot S6 Universal Reader (CTL). The final determinations were calculated by subtracting the negative stimulation background levels from the measured values.

### Quantification of tissue viral load

The pulmonary live virus represent in mouse was tested using a focus reduction neutralization test by the State Key Laboratory of Respiratory Disease in Guangdong (29). At the dedicated times shown in **Figure 2A**, specimens of swab, trachea, bronchus, lung LN, BALFs, and six lung lobes were collected for viral RNA detection. Tissues were weighed, homogenized, and clarified by centrifugation at 8,000 rpm for 10 min at 4°C, and the supernatants were obtained. Viral RNA was extracted using a magnetic viral nucleic acid kit (TIANGEN, China), according to the manufacturer’s protocol. The viral gRNA in each sample was quantified using reverse transcription PCR (RT-PCR) targeting the ORF1ab and N genes of SARS-CoV-2. For sgRNA quantification, RT-PCR was performed targeting the sequence overlapping the ORF1ab and E genes of SARS-CoV-2. The LLOD of viral RNA was set at 1 copy/μl because of the detection limits of this assay. The LLOD was transformed to 2500 copies/g for the lung and 500 copies/ml for BALF and swabs according to the volume of the RNA extraction reagent.

### Histopathology

Lung pathology was evaluated using hematoxylin and eosin (H&E) staining, as previously described (30). Five-micron-thick formalin-fixed paraffin-embedded (FFPE) sections were prepared and stained. The slides were evaluated by a pathologist in a double-blinded manner. To prepare ultrastructural sections, tissues were embedded in epoxy resin and cut into 60–70 nm sections. Pathological scores were calculated as described by Liu et al. (31).

### Multiple cytokine profiling

Lung lobes and bronchoalveolar lavages were collected at day 7 post-challenge. Lung lobe specimens were weighed, and the supernatants of the homogenates were prepared for detection. Pro-inflammatory cytokines, including IL-1β, IL-6, TNF-α, IFN-γ, IL-8, G-CSF, GM-CSF, MCP-1, MIP-1β, and MIP-1β, in all supernatants and BALFs were measured using the MILLIPLEX MAP Non-Human Primate Cytokine Magnetic Bead Panel - Immunology Multiplex Assay. The concentration of each sample was calculated using a standard curve.

### Statistical analysis

Antibody titers were transformed into log_10_ values to calculate the GMTs. All statistical analyses were performed using GraphPad Prism v.7.0 (GraphPad Software, Inc.). Comparisons between two groups were performed using two-tailed t-tests, whereas those among multiple groups were performed using one-way analysis of variance (ANOVA). A P value of < 0.05 was considered statistically significant.

## Results

### Immune responses and protective efficacy induced by heterologous and homologous immunization with ChAd-S and IV in mice

**Figure 1:**
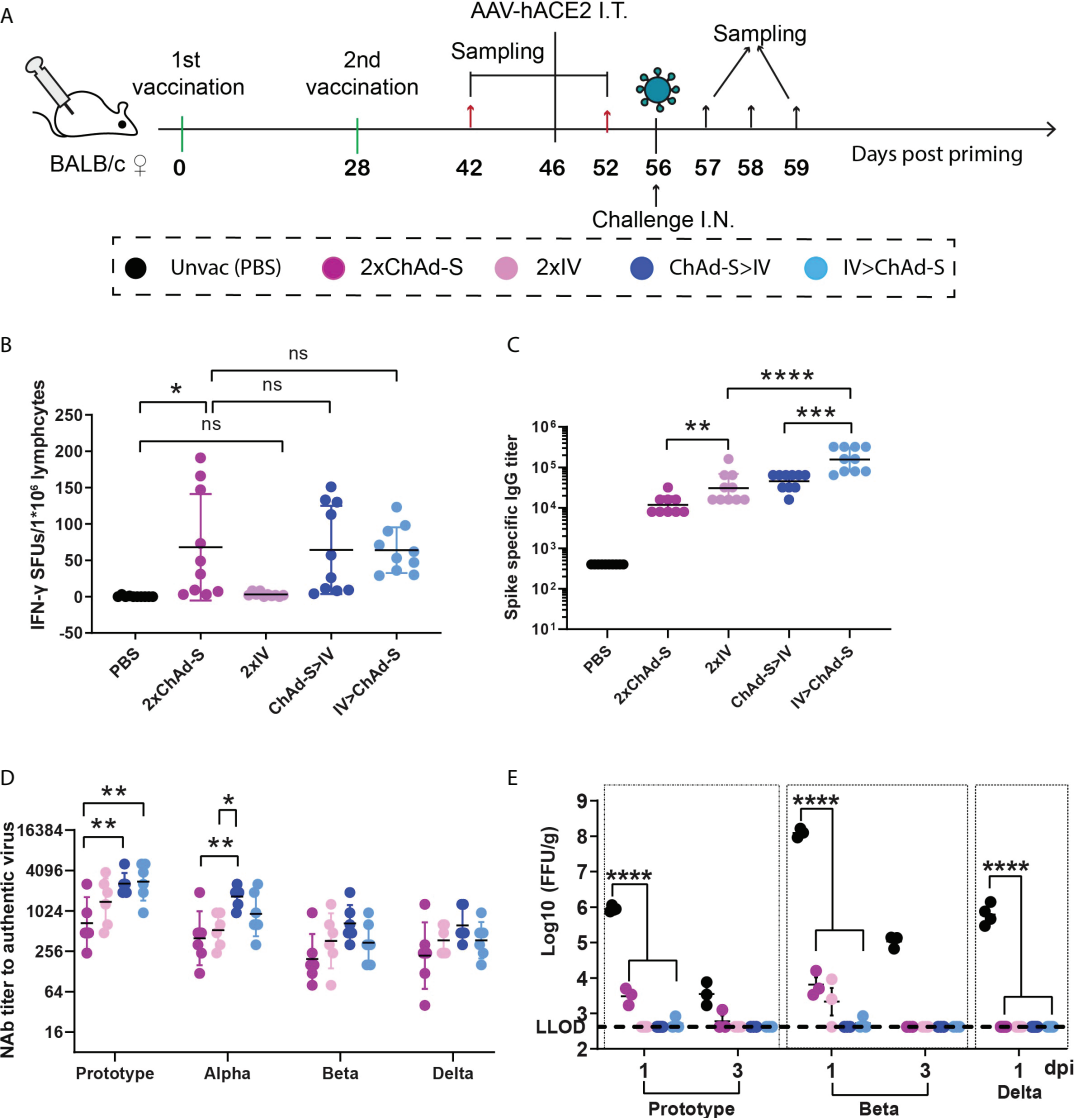
Immune responses and protective effect induced by homologous or heterologous prime-boost with ChAd-S and IV in mice **(A)**. Schematic representation of experimental protocol. Mice in 4 groups were immunized with different vaccines: 2×ChAd-S, 2×IV, ChAd-S>IV, IV>ChAd-S. Unvac group were injected with PBS. N=16 per group. **(B)** Splenocytes on day 42 were stimulated with spike peptide pool, and the IFN-γ secreting cells were quantified by ELISPot assay and expressed as spot forming units (SFU). N=10 per group. **(C)** Serum spike specific IgG titers were measured by ELISA on day 42. N=10 per group. **(D)** Serum NAb titers measured by live SARS-CoV-2 virus were expressed as 50% inhibitory dilution (EC50) of serum. N=6 per group. **(E)**. Lung viruses were quantified by a focus reduction neutralization test and expressed as FFU/g. N=6 per group, one spot represents one animal. One-way ANOVA was performed for **(B–E)** Bars represent the geomean ± geometric SD for c and d, mean± SD for **(B, E)** ns: p>0.05, *p<0.05, **p<0.01, ***p<0.001, ****p<0.0001.

To evaluate the immunogenicity of ChAd-S and IV, and to optimize the immunization strategy with the two vaccines, BALB/c mice were randomly divided into four groups with 16 mice per group and vaccinated on days 0 and 28 with two doses of ChAd-S (2×ChAd-S), two doses of IV (2×IV), one dose of ChAd-S and boosted with IV (ChAd-S >IV), one dose of IV and boosted with ChAd-S (IV> ChAd-S), respectively (**Figure 1A**). The dosage was 1×10^10^ vp for ChAd-S and 1μg for IV. On day 42, 10 mice from each group were sacrificed for spike-specific IgG and T-cell response measurements. For the T cell response test, splenocytes were stimulated by a pool of peptides spanning the full-length spike, and an enzyme-linked immunospot (ELISPot) assay was conducted to quantify INF-γ-secreting cells. The 2×ChAd-S, ChAd-S>IV, and IV>ChAd-S groups showed robust and comparable T-cell responses, with average spot-forming units (SFUs) of 67.9, 63.9, and 64.2, respectively. Two doses of IV failed to elicit spike-specific T-cell responses (**Figure 1B**). For the spike-specific IgG titer, the 2×IV group had a geometric mean titer (GMT) of 30530, which was slightly higher than that of the 2×ChAd-S group (GMT=11757). The IV>ChAd-S and ChAd-S>IV groups showed a further increased level of binding IgG titer (by 5.12-fold and 1.48-fold) compared with that of the 2×IV group. Specifically, the GMT in group IV>ChAd-S was 156469, which was 3.46-fold higher than that in the ChAd-S>IV group (**Figure 1C**).

To evaluate the protective efficacy of the four vaccination regimens in mice, the remaining six immunized mice from each group were treated with 2×10^11^ genome copies of adeno-associated virus expressing hACE2 on day 46 to construct a temporary hACE2-transgenic mouse model and then subjected to a challenge experiment on day 56 (**Figure 1A**). Neutralizing antibody (NAb) titers have been reported to be an important correlate of COVID-19 vaccine-induced protection (CoP) (32, 33). Thus, the level of serum cross-reactive NAb titers for each group was measured before the challenge on day 52. The results showed that the NAb GMTs of group 2×IV were 1396, 528, 366, and 373 for the prototype, Alpha, Beta, and Delta strains, respectively, which were slightly higher than those of the 2×ChAd-S group with NAb GMTs of 634, 400, 196, and 220. The ChAd-S>IV and IV>ChAd-S regimens further improved the NAb titers compared with those of single ChAd-S or IV regimen. Specifically, ChAd-S>IV showed higher NAb GMT than the IV>ChAd-S group and elevated NAb titers by 4.11- (p=0.0091), 4.19- (p=0.0089), 3.39- (p=0.0676), and 2.82-fold (p=0.1125) for the prototype, Alpha, Beta, and Delta strains compared with those of the 2×ChAd-S regimen, and 1.87- (p=0.4075), 3.17- (p=0.0404), 1.81- (p=0.5771), and 1.67-fold (p=0.6519) higher than those of the 2×IV regimen (**Figure 1D**). These data indicate that the heterologous prime-boost strategy can effectively improve antigen-specific cellular and humoral responses.

On day 56, mice were subjected to intranasal challenge with the prototype, Beta (B.1.351), and Delta (B.1.617.2) SARS-CoV-2 strains using 5×10^4^ focus forming units (FFU) (**Figure 1A**). For the prototype and Beta challenge, lung samples were collected at days 1 and 3 post-infection (dpi). For the Delta challenge, the lung samples were collected at 1 dpi. Lungs were subjected to viral titer quantification using the focus reduction neutralization test (FRNT). Lung viruses were undetectable in the ChAd-S>IV and IV>ChAd-S groups at days 1 and 3 after challenge with any of the three VOCs. For the homologous vaccine regimens, the 2×ChAd-S regimen group showed 3066 FFU/g virus after the prototype strain challenge and 6465 FFU/g virus for the Beta strain challenge at 1 dpi, while the 2×IV group showed 2138 FFU/g virus in the lung for the post-strain challenge. These data demonstrated that heterologous prime-boost with ChAd-S and IV enhanced the protective immune response and protection against diverse SARS-CoV-2 variants in mice. Among these, regimen priming with ChAd-S and boosting with IV was prioritized.

### Immune responses induced by the heterologous regimen in rhesus macaques

**Figure 2:**
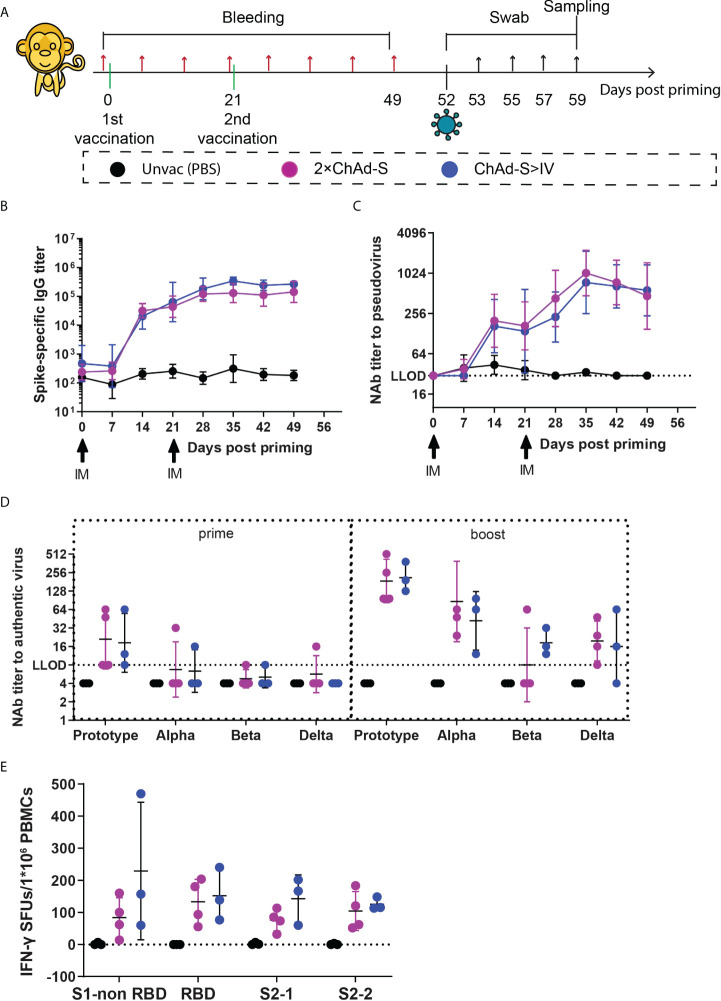
Immune responses induced by homologous ChAd-S or heterologous ChAd-S plus IV in NHPs **(A)**. Schematic representation of the experimental protocol. Female rhesus macaques were immunized with 2 doses of ChAd-S (2×ChAd-S, n = 4), or prime with ChAd-S and boost with IV (ChAd-S>IV, n = 3). “Unvac” was unvaccinated group and set as the blank control. For all NHPs, blood was collected before immunization and every 7 days post priming. On day 52, NHPs were challenged with Beta variant (B.1.351). Nasal swabs and throat swabs were collected at dedicated time point as shown, and all NHPs were sacrificed on day 7 post challenge for trachea, bronchus, lung, and BAL. **(B–D)** Humoral immune responses, including the dynamic titer of sera spike protein-specific IgG **(B)**, dynamic NAb titer measured by pseudovirus **(C)**, and cross-neutralizing antibody titer against Prototype, Alpha, Beta and Delta **(D)**. NAb titers were expressed as 50% effective dilution (ED50) of serum. **(E)** Spike-specific T cell responses of PBMC were measured by IFN-γ ELISPot assay, and expressed as SFU per 1×10^6^ lymphocytes. Bars represent the geomean ± geometric SD for **(B–D)**, mean± SD for **(E)**.

Since heterologous immunization with ChAd-S plus IV effectively improved humoral immune responses compared with those of the homologous vaccination group, and induced equivalent spike-specific T cell responses in the single ChAd-S group in mice, we next tested the ChAd-S >IV and 2×ChAd-S regimens in young rhesus macaques. Ten female NHPs aged 2-3 years old were randomly divided into three groups: three in the unvaccinated control group, four in the 2×ChAd-S group, and three in the ChAd-S >IV group. The unvaccinated control group was shared with another study conducted by our group (34). For macaque experiments, all NHPs were administered 0.5×10^11^ vp for ChAd-S and 5μg for IV. For the 2×ChAd-S group, NHPs were immunized intramuscularly with two doses of ChAd-S at an interval of 21 days. For the ChAd-S>IV group, NHPs were immunized with one human dose of ChAd-S followed by one dose of IV on day 21 post-priming. Serum samples were collected every seven days for antibody detection, and peripheral blood mononuclear cells (PBMCs) were isolated for spike-specific T cell quantification using an interferon-γ (IFN-γ) ELISPot assay (**Figure 2A**). In our study, serum spike-specific IgG and pseudovirus neutralizing antibody in all vaccinated macaques converted positive 14 days after ChAd-S priming, with a GMT of 26397 for binding antibodies and 183 for pseudovirus neutralizing antibodies. The antibody GMTs of the 2×ChAd-S and ChAd-S>IV groups both increased after the second dose and peaked at day 35 with GMTs of 13133 and 343351 for binding antibodies, and 887 and 743 for pseudovirus neutralizing antibodies, respectively (**Figures 2B, C**). No significant differences in the binding and pseudovirus antibody titers were detected between the 2×ChAd-S and ChAd-S>IV groups.

Furthermore, the level of cross-neutralizing antibodies was measured against the authentic virus on day 21 (three weeks post-priming) and 49 (four weeks post-boosting) to evaluate the potential neutralizing ability of NHP sera. Although all NHP sera in the vaccine groups converted positive against the prototype strain at day 7 after ChAd-S priming, the neutralizing reactivity was poor with a GMT of 19.84 for the seven NHPs from the 2×ChAd-S and ChAd-S>IV groups. The levels of NAb GMT to the prototype strain were highly improved to 186.43 for the 2×ChAd-S group and 211.34 for the ChAd-S>IV group (**Figure 2D**). For reactivity against different VOCs, the NAb GMT in the ChAd-S>IV group decreased to 41.93 for the Alpha, 18.32 for the Beta, and 16.00 for the Delta strains. Similarly, the NAb GMT in the 2×ChAd-S group decreased to 86.75 for the Alpha, <8.00 for the Beta, and 19.59 for the Delta strains (**Figure 2D**). Thus, the Beta and Delta strains were more resistant to vaccinated NHP serum. Interestingly, the NAb GMT against the Beta strain in the ChAd-S>IV group was slightly higher than that in the 2×ChAd-S group. Specifically, the NAb GMT in three out of four NHPs in the 2×ChAd-S group dropped below the low limit of detection (LLOD), while that of NHPs in the ChAd-S>IV group was higher and ranged from 12 to 64.

Spike-specific T cell responses were also measured before the SARS-CoV-2 challenge in this experiment. PBMCs were isolated from blood samples at day 49 and stimulated with four separate pools of peptides spanning the full-length spike. Robust T cell responses were elicited for all NHPs in the vaccinated groups. The spike specific IFN-γ SFUs/1×10^6^ PBMCs in the ChAd-S>IV groups ranged from 125.56 to 228.89 for the four peptide pools and were slightly higher but not significantly different from those in the 2×ChAd-S group (**Figure 2E**).

### Heterologous immunization with ChAd-S plus IV inhibited pulmonary viral replication in macaques

**Figure 3:**
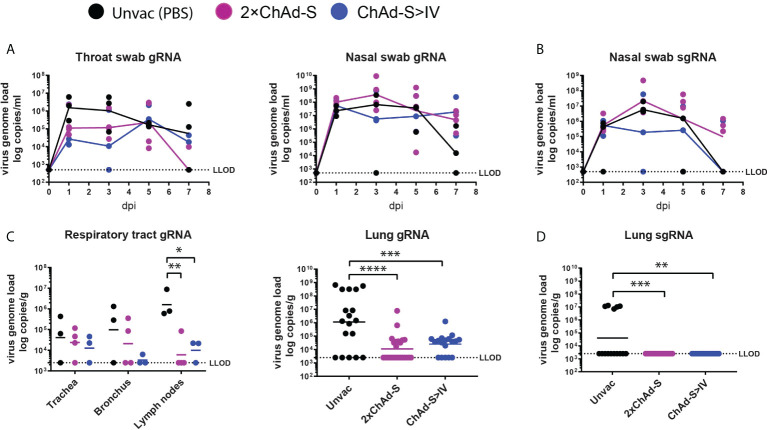
Viral load in NHPs after challenge by Beta variant. Throat swabs, nasal swabs, trachea, bronchus, lung lymph nodes, and lung lobe specimens were collected at the time shown in **Figure 1A**. **(A–D)** SARS-CoV-2 gRNA and sgRNA levels were measured by RT-PCR and expressed as viral copies. LLOD was the low limit of detection. gRNA was quantified in throat swabs (left panel) and nasal swabs (right panel) **(A)**, trachea, bronchus, lung lymph nodes (left panel) and six lung lobes (right panel, one spot represent one lung lobe out of six lobes for each animal) **(C)**. sgRNA was quantified in nasal swabs **(B)** and six lung lobes **(D)**. One spot represents one animal. One-way ANOVA was performed **(C, D)** *p<0.05, **p<0.01, ***p<0.001, ****p<0.0001.

The Beta variant was shown to be more difficult to neutralize than the Alpha and Delta variants in the immunized serum. Thus, we selected the Beta variant for the NHP challenge experiment to test the potential protective effect of the prime-boost regimen against SARS-CoV-2 infection (**Figure 2A**). Nasal and throat swabs were collected every two days post-challenge for dynamic viral load quantification. No significant reduction in the levels of swab genomic RNA (gRNA) was found in vaccinated groups compared with those in unvaccinated groups (**Figure 3A**). Macaques were euthanized and necropsied seven days post-challenge, and viral loads were tested in the trachea, bronchus, lung lymph nodes (lung LN), and lung lobes. The mean level of gRNA copies of SARS-CoV-2 in vaccinated groups was reduced in the trachea, bronchus, lung LN, and six lung lobes compared with that in the unvaccinated group. Specifically, the average number of gRNA copies in the ChAd-S>IV group in the trachea (ChAd-S>IV:13667, 2×ChAd-S: 23538) and bronchus (ChAd-S>IV: 3399, 2×ChAd-S: 20806) was lower than that in the 2×ChAd-S group, while that in the lymph nodes was slightly higher (ChAd-S>IV: 10564, 2×ChAd-S: 6015) (**Figure 3C**, left panel). The average viral loads in the six lung lobes were comparable between the two vaccinated groups (p=0.6552) (**Figure 3C**, right panel). Nasal swabs and lung samples were tested for sub-genomic RNA (sgRNA) to evaluate viral replication in the respiratory tract and lungs. The viral sgRNA level in the vaccinated and unvaccinated groups peaked at 2-3 dpi and then gradually decreased. No significant reduction in the swab viral sgRNA level was found in the vaccinated group compared with that in the unvaccinated group (**Figure 3B**). For lung tissue, viral sgRNA was only detected in the lung lobes of the unvaccinated group, but not in those of the vaccinated group, indicating that the 2×ChAd-S and ChAd-S >IV vaccination regimens successfully inhibited viral replication (**Figure 3D**).

### Heterologous immunization with ChAd-S plus IV reduced the pulmonary histopathologic changes and levels of inflammatory cytokines in macaques

**Figure 4:**
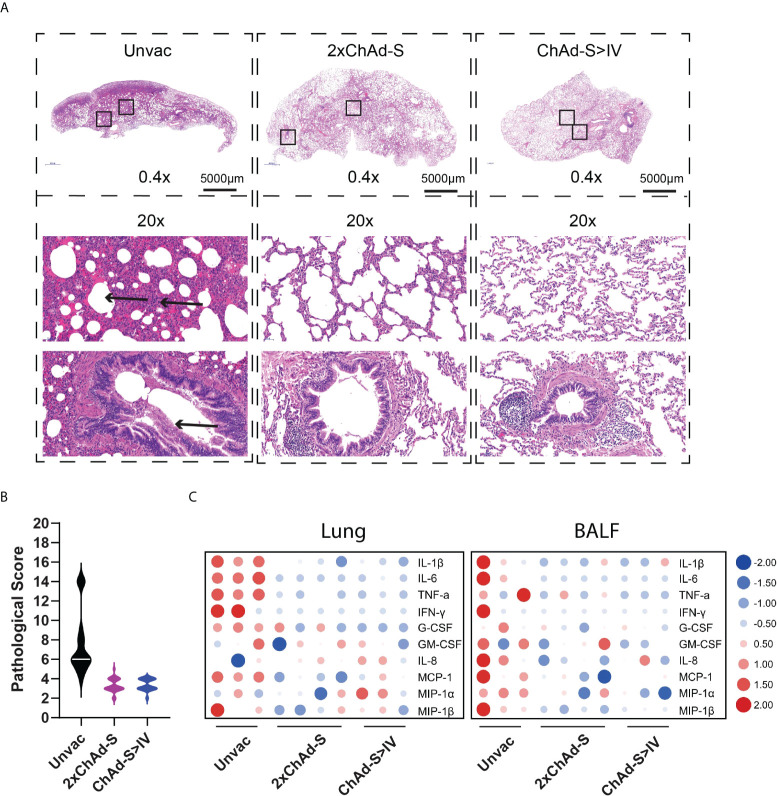
Pulmonary histopathology and inflammatory cytokine analysis of NHPs after challenge. **(A)** Representative graph of hematoxylin and eosin (H&E) staining of lung tissues. Arrowhead indicates the histopathological lesion. **(B)** Pathological score of all lung lobes of each macaque (18 specimens for each group) was counted according to the severity and extent of histopathological lesion and represented as violin plot. **(C)** The concentrations of multiple inflammatory cytokines in lung homogenate (left panel) and BALs (right panel) in different groups were compared using heatmap. The concentration of each cytokine was transformed by log2 and normalized by Z-score.

The histopathological changes in the vaccinated and unvaccinated macaques were assessed. As previously reported, moderate to severe interstitial pneumonia characterized by inflammation, thickened adjacent alveolar interstitial, expanded alveolar spaces, and sloughing of epithelial cells in the distal bronchioles was observed in the unvaccinated group. The alveolar spaces of one macaque in the unvaccinated group were filled with homogenous eosinophilic proteinaceous fluid. In contrast, the pathological changes in the vaccinated groups were much less severe (**Figure 4A**), with an average pathological score of 3.30 in the 2×ChAd-S group and 3.39 in the ChAd-S>IV group, which was lower than the score of 7.50 in the unvaccinated group (**Figure 4B**). Thus, both the 2×ChAd-S and ChAd-S>IV regimens effectively alleviated lung pathology.

Hyperproduction of pro-inflammatory cytokines is associated with alveolar injury in COVID19, and is further associated with severe illness and death. To investigate the lung inflammation status in vaccinated macaques, lung homogenates and bronchoalveolar lavage fluids (BALFs) were subjected to multi-cytokine quantification. As a result, the levels of general pro-inflammatory cytokines, such as interleukin-1β (IL-1β), interleukin-6 (IL-6), tumor necrosis factor-α (TNF-α), and IFN-γ, in the lungs and BALF were reduced in vaccinated groups compared with those of the unvaccinated group (**Figure 4C**).

### Immune activation in blood after infection

**Figure 5:**
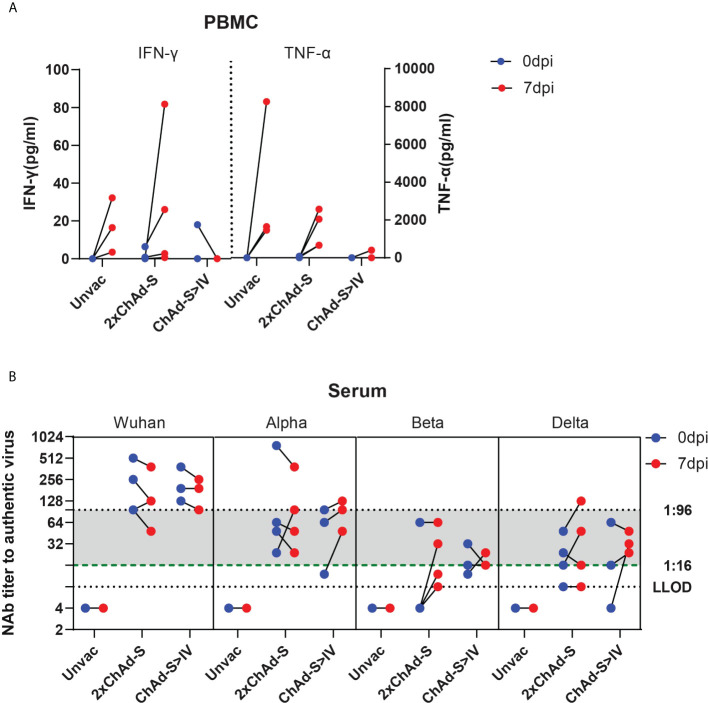
Recalled immune responses after challenge in different vaccine groups. **(A)** PBMCs were isolated before (0 dpi) and after (7 dpi) challenge, and stimulated by spike peptide pool. INF-γ concentration of supernatant was quantified and expressed as pg/ml. **(B)** Serum neutralizing antibody titer against different VOCs before (0 dpi) and after (7 dpi) Beta variant challenge. One spot represents one animal.

To characterize the immune responses recalled by infection in heterologous and homologous vaccinated NHPs, the blood T cell and NAb responses were tested before and after the viral challenge. For the spike-specific T cell responses, PBMCs at 0 and 7 dpi were isolated from blood and stimulated with the full-length spike peptide pool, and the IFN-γ levels were measured in the supernatants. As a result, the concentration of secreted IFN-γ was evidently increased by 3.55-16.55 pg/ml in the unvaccinated group and 1.94-19.96 pg/ml in the 2×ChAd-S group after the viral challenge, whereas no increase was found in the ChAd-S>IV group **Figure 5A**. Similar to INF-γ, the TNF-α level was highly elevated in the unvaccinated group and the 2×ChAd-S group by 1458.38-8263.83 pg/ml and 640.88-2568.30 pg/ml, respectively. However, only one macaque in the ChAd-S>IV group manifested an elevation of the TNF-α level at a far lower level than that in the other groups **Figure 5B**. These results suggest that the T cell responses in the ChAd-S>IV group were relatively lower than those in the single ChAd-S regimen, indicating a lower risk of pulmonary immune injury.

The serum NAb titers at 0 and 7 dpi were tested against the prototype, Alpha, Beta, and Delta strains. We found that sera with an NAb titer under 1:16 against specific VOCs at 0 dpi evidently increased at day 7 after challenge, whereas those higher than 1:96 maintained a naturally declining trend. For sera with NAb titers between 1:16 and 1:96, the NAb titer increased or decreased. This result suggests that the recalled NAb responses depended on whether the level of pre-existing NAb was sufficient to clear the virus.

### Additional heterologous or homologous booster strengthened and prolonged the anti-SARS-CoV-2 immunity in mice

**Figure 6:**
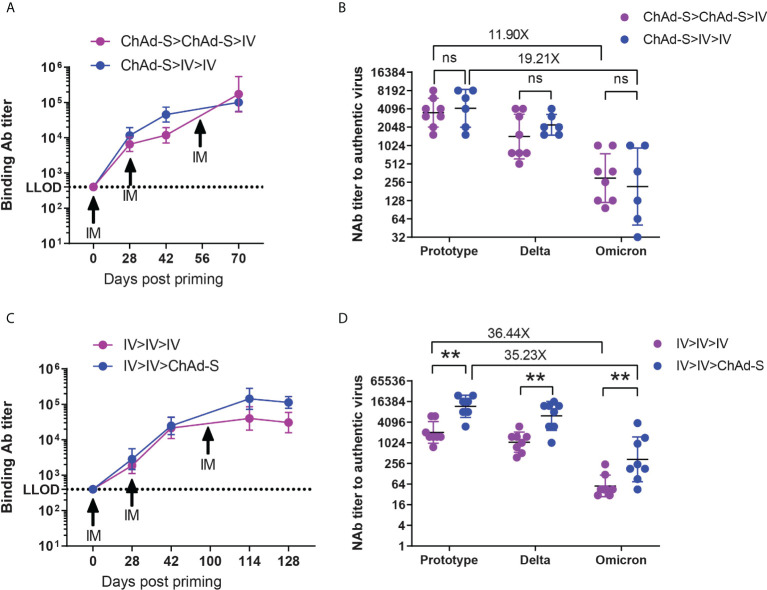
The humoral immune responses induced by additional heterologous or homologous booster in mice. **(A, B)** Mice immunized with 2×ChAd-S and ChAd-S>IV on day 0 and 28 were further boosted by ChAd-S on day 56. Serum spike specific IgG titers were measured by ELISA on day 0, 28, 42 and 70 **(A)**, and NAb titers against Delta and Omicron were measured on day 70 and expressed as 50% inhibitory dilution (EC50) of serum **(B)**. **(C, D)**. Mice immunized with 2×IV on day 0 and 28 were further boosted by IV or ChAd-S on day 100. Serum spike specific IgG titers were measured by ELISA on day 0, 28, 42, 114 and 128 **(C)**, and NAb titers against Delta and Omicron were measured on day 114 and expressed as 50% inhibitory dilution (EC50) of serum **(D)**. N=9 per group. Student’s t test was performed for b and d. Bars represent the geomean ± geometric SD, ns: p>0.05, **p<0.01.

To further improve the immunity induced by the two basic doses of COVID-19 vaccines, mice were immunized with 2×ChAd-S or ChAd>IV, as shown in **Figure 1**, and all mice were boosted using a third dose of IV at day 28 after the second dose, shown as ChAd-S>ChAd-S>IV and ChAd-S>IV>IV group (**Figure 6**). The serum spike-specific IgG levels at day 14 after the second and third doses were measured and compared. The additional dose of IV significantly increased the serum spike-specific IgG levels compared with those of the two doses of basic vaccination (**Figure 6A**). The GMT of spike-specific IgG and NAb against the Prototype, Delta and Omicron strains was comparable between ChAd-S>ChAd-S>IV group (3547 for prototype, 1438 for Delta, 298 for Omicron) and ChAd-S>IV>IV group (4177 for prototype, 2234 for Delta, 217 for Omicron) (**Figures 6A, B**). IVs have been administered to a large human population, and the immunity against the Delta and Omicron strains was reported to be significantly lower than that against the prototype. We tracked the serum IgG induced by two doses of IV for a long time in mice and attempted an additional booster dose with the homologous IV or heterologous ChAd-S vaccine. As a result, the additional dose of IV or ChAd-S could further increase the serum spike-specific IgG levels, whereas the additional heterologous ChAd-S boost was more effective with higher levels of IgG GMT and NAb titers against the Prototype, Delta and Omicron strains (**Figure 6C**). To be specific, the GMT of NAb titers were 2065 for Prototype, 1070 for Delta, 57 for Omicron in IV>IV>IV group and 11881 for prototype, 6276 for Delta, 337 for Omicron in IV>IV>ChAd-S group (**Figures 6C, D**). These results demonstrated that the additional third dose of heterologous or homologous vaccine could strengthen and prolong the anti-SARS-CoV-2 immunity in a mouse model, however, a higher antibody titer was achieved using the heterologous boost strategy.

## Discussion

To enhance the general immune response to different kinds of COVID-19 vaccines, we tried a heterologous prime-boost strategy with ChAd-S and IV in mouse and rhesus macaque models. The ChAd-S>IV and IV>ChAd-S regimens showed higher NAb titers compared with those of the two doses of single ChAd-S or IV in mice. Among them, the ChAd-S>IV regimen induced higher NAb titers than the IV>ChAd-S regimen, indicating that the heterologous prime-boost strategy could effectively improve the immunogenicity of the two aforementioned vaccines. The induction of robust T cell responses was shown to be a distinct feature of the ChAd vectored vaccine, as previously reported (35). The frequency of IFN-γ-secreting T cells elicited by an exogenous IV was significantly elevated by the combined vaccination regimen with the ChAd vectored vaccine. This is consistent with our previous studies on a human adenovirus type-5 vectored vaccine, in which heterologous immunization with this vaccine improved the T cell responses to an IV and protein subunit vaccine (21). In addition to the direct elimination of infected cells, activated T cells provide essential support for B cell differentiation and antibody affinity maturation (36). Moreover, T cells that recognize conserved short-linear peptides could be a potential immune arm for resisting mutated SARS-CoV-2 (17, 37, 38). Therefore, it is rational to combine ChAd-S with IVs for broader immunity.

The compatibility and protective efficacy of the combined vaccination regimens were tested in rhesus macaques. The results showed that both the ChAd-S>ChAd-S and ChAd-S>IV regimens effectively induced neutralizing activity and Th1 cell responses against the prototype strain. Nevertheless, inconsistent with what was found in the mouse model, no significant difference in immune responses was found between the ChAd-S>ChAd-S and ChAd-S>IV. The different observations in different animal models might be due to the small sample size, more complex background, and larger inter-individual differences in macaques, or due to the greater coefficient of variation of biological detection methods (39). From the data in mice and macaques, it could be concluded that heterologous prime-boost with ChAd vectored vaccine and IV optimized and modulated the protective immune responses, or at least did not reduce them.

The recalled immune responses should be sufficient to defend against SARS-CoV-2 invasion, whereas over-activation of the cytotoxic T cell response might initiate lung injury (40). Hyper production of IFN-γ by Th1 cells during infection will activate macrophages and TNF-α as well as chemokines, which might finally cause tissue inflammation and damage (41). The 2×ChAd-S immunization protocol resulted in a comparable level of specific secretion of IFN-γ and TNF-α by PBMCs after the viral challenge in the unvaccinated group, indicating a sensitive recall response of vaccine induced T cell but also higher risk of T cell-induced lung injury. Interestingly, the specific recalled T-cell response in the ChAd-S>IV group was evidently lower. This suggests that heterologous prime-boost with ChAd-S plus IV might prevent the over-activation of pathogenic immune responses after SARS-CoV-2 infection. Meanwhile, the NAb responses in the ChAd-S>IV group were not affected by the relatively lower T cell responses but were highly dependent on the pre-existing NAb levels. A high level of pre-existing NAbs might be sufficient to inhibit viral replication at the initial stage of infection; thus, the antibody response would not call for an additional upregulation to fight the virus. However, for those with antibody levels lower than 1:16 additional upregulation is quickly recalled to confer a similar level of protection. This has also been confirmed in our previous study (34).

The NAb levels against different VOCs induced by homologous or heterologous prime-boost strategies were reduced by various degrees due to mutations in the receptor-binding domain (RBD), which has been confirmed in several other studies (42, 43). The E484K mutation is a dominant cause of the reduction of neutralizing activity against the Beta variant (44), and are also present in the currently circulating VOCs, including the Delta and newly defined Omicron. As demonstrated in this study, the level of serum NAb titers against the Beta variant was lower than that against the prototype and Alpha strains, and slightly lower than that of the Delta strain in both mice and macaques. Therefore, we selected the Beta strain with a relatively greater immune evasion ability to challenge macaques to better evaluate and explain the protective potential of the vaccination strategies against different mutant strains. Although 11.5-fold and over 23.3-fold reductions in the NAb titers against the Beta strain were induced by the ChAd-S>IV and 2×ChAd-S regimens, respectively, they still mediated effective protection against the immune escape Beta variant in NHPs with significant reduction of the viral load, pathogenic manifestation, and inflammatory cytokine levels in the respiratory system. The serum antibodies induced by the ChAd-S>IV and 2×ChAd-S regimens still effectively recognized the Alpha and Delta strains, and the titer was higher than that against the Beta strain. As NAbs have been proven to be a definite CoP for COVID-19, these results might predict that the aforementioned homologous and heterologous prime-boost strategies could also confer potential protection against the prototype, Alpha, and Delta strains in macaques. Furthermore, though no significant difference shown in anti-S IgG or neutralizing antibody titers in NHP immunized with 2×ChAd-S or with ChAd-S>IV, the heterologous vaccine (ChAd-S>IV) appeared to reduce the activation of IFN-γ producing Th1 T cells compared to the 2×ChAd-S vaccine post infection, which might be an index of reduction of pulmonary inflammation (**Figure 5A**). Thus, a multi-dose heterologous inactivated virus vaccine might reduce the adverse reactions induced by multi-dose homologous virus vector-based vaccine and benefit large-scale population vaccination. However, the currently circulating Omicron variants contain a large number of key mutations, with 32 mutations in the spike protein, which were shown to be more difficult to neutralize by vaccinated serum compared to previously reported variants. Additional doses of booster vaccines or new vaccines targeting Omicron are urgently required to maintain population immunity against SARS-CoV-2.

The immune effect of an additional third-dose vaccination was investigated to further improve and prolong vaccine-mediated protection against all VOCs. First, mice vaccinated with two IV doses were further boosted with an additional dose of homologous IV or heterologous ChAd-S vaccine. As a result, both homologous IV and heterologous ChAd-S boosting after the two doses of IV further improved the antibody responses. However, heterologous boosting with ChAd-S was superior to homologous IV boosting in spike-specific antibody induction (**Figure 5B**). Recently, a phase 4 clinical trial was conducted to verify the immunogenicity of heterologous and homologous boost regimens after two doses of the inactivated vaccine CoronaVac; similarly, heterologous boosting with ChAd or Ad26 vectored vaccines resulted in more robust immune responses compared with homologous IV boosting (45). Meanwhile, mice that completed the 2×ChAd-S or ChAd-S>IV regimen were further boosted with IV in this study. Although the serum spike-specific IgG GMT in the 2×ChAd-S group was relatively lower than that in the ChAd-S>IV group after the second vaccine dose, it drastically increased and overtook the ChAd-S>IV group after a third dose of heterologous IV boost. Interestingly, an additional third vaccine dose prolonged the NAb responses and achieved effective neutralizing activity against the highly immune escape Omicron variant. Together with data from two-dose prime-boost vaccinations, our data demonstrated that heterologous boost at the second dose and the additional third dose might be a more effective approach to enhance antibody responses. Nonetheless, the experimental model used in this study could not fully reflect the real circumstances in humans, and more clinical studies are still needed to assure the compatibility between divergent vaccine platforms.

In summary, to improve vaccine-induced immunity, we tested a homologous and heterologous prime-boost strategy using a chimpanzee adenovirus vectored vaccine and an IV in mice and macaques. The heterologous prime-boost regimen with ChAd-S and IV maintained or elevated the antibody and T cell responses compared with those of each single vaccine regimen and mediated effective protection against immune escape variants. Interestingly, the heterologous ChAd>IV regimen might prevent immune activation in vaccinated NHPs after viral challenge because of lower T cell responses but comparable cross-reactive NAb responses. This study also analyzed the correlation between pre-existing NAb levels and recalled NAb responses. Our study demonstrated the good compatibility of chimpanzee adenovirus vectored vaccines and IV in animal models.

## Data availability statement

The raw data supporting the conclusions of this article will be made available by the authors, without undue reservation.

## Ethics statement

The animal study was reviewed and approved by the Institutional Animal Care and Use Committee of the National Institutes for Food and Drug Control, China the Institutional Animal Care and Use Committee at the State Key Laboratory of Respiratory Disease in Guangdong the Institutional Animal Care and Use Committee of the Institute of Medical Biology, Chinese Academy of Medicine Sciences & Peking Union Medical College, China. Written informed consent was obtained from the owners for the participation of their animals in this study.

## Author contributions

JW, MX, and ZL conceived the study. QH and QM designed the study. QH wrote and revised the manuscript. QH, JLZ and FG performed all the experiments. QH, QM, FG, LB, and XW analyzed the data. YB, BC, JL, CA, QW, XY, JY, LS, ZS, DL, YY, JS and JCZ helped in the experiments and data analysis WH and CL provided some materials. All authors reviewed and approved the final manuscript.

## Funding

This work was supported by the Emergency Key Program of Guangzhou Laboratory (No. EKPG21-30-1 and No. EKPG21-28) and the National Key R&D Program of China (No. 2021YFC2301700), and Guangdong Basic and Applied Basic Research Foundation (2022B1515020059).

## Conflict of interest

The authors declare that the research was conducted in the absence of any commercial or financial relationships that could be construed as a potential conflict of interest.

## Publisher’s note

All claims expressed in this article are solely those of the authors and do not necessarily represent those of their affiliated organizations, or those of the publisher, the editors and the reviewers. Any product that may be evaluated in this article, or claim that may be made by its manufacturer, is not guaranteed or endorsed by the publisher.

## References

[1] WHO. COVID-19 vaccine tracker and landscape. Available at: https://www.who.int/publications/m/item/draft-landscape-of-covid-19-candidate-vaccines.

[2] WHO. Criteria for COVID-19 vaccine prioritization. Available at: https://www.who.int/publications/m/item/criteria-for-covid-19-vaccine-prioritization.

[3] WHO. WHO coronavirus (COVID-19) dashboard. Available at: https://covid19.who.int/.

[4] Widge AT Rouphael NG Jackson LA Anderson EJ Roberts PC Makhene M. Durability of responses after SARS-CoV-2 mRNA-1273 vaccination. N Engl J Med (2021) 384:80–2. doi: 10.1056/NEJMc2032195 PMC7727324 33270381

[5] Favresse J Bayart JL Mullier F Elsen M Eucher C Van Eeckhoudt S. Antibody titres decline 3-month post-vaccination with BNT162b2. Emerg Microbes Infect (2021) 10:1495–8. doi: 10.1080/22221751.2021.1953403 PMC8300930 34232116

[6] Khoury J Najjar-Debbiny R Hanna A Jabbour A Abu Ahmad Y Saffuri A. COVID-19 vaccine - long term immune decline and breakthrough infections. Vaccine (2021) 39:6984–9. doi: 10.1016/j.vaccine.2021.10.038 PMC8556595 34763949

[7] Pegu A O'Connell SE Schmidt SD O'Dell S Talana CA Lai L. Durability of mRNA-1273 vaccine-induced antibodies against SARS-CoV-2 variants. Sci (New York NY) (2021) 373:1372–7. doi: 10.1126/science.abj4176 PMC8691522 34385356

[8] Edara VV Norwood C Floyd K Lai L Davis-Gardner ME Hudson WH. Infection- and vaccine-induced antibody binding and neutralization of the B.1.351 SARS-CoV-2 variant. Cell Host Microbe (2021) 29:516–21.e3. doi: 10.1016/j.chom.2021.03.009 33798491 PMC7980225

[9] Cele S Jackson L Khoury DS Khan K Moyo-Gwete T Tegally H. Omicron extensively but incompletely escapes pfizer BNT162b2 neutralization. Nature (2022) 602:654–6. doi: 10.1038/s41586-021-04387-1 PMC8866126 35016196

[10] Chen J Wang R Gilby NB Wei GW. Omicron (B.1.1.529): Infectivity, vaccine breakthrough, and antibody resistance. ArXiv (2021) 62(2):412–422. doi: 10.1021/acs.jcim.1c01451 PMC8751645 34989238

[11] Excler JL Kim JH. Novel prime-boost vaccine strategies against HIV-1. Expert Rev Vaccines (2019) 18:765–79. doi: 10.1080/14760584.2019.1640117 31271322

[12] Lu S. Heterologous prime-boost vaccination. Curr Opin Immunol (2009) 21:346–51. doi: 10.1016/j.coi.2009.05.016 PMC3743086 19500964

[13] Wang S Parker C Taaffe J Solórzano A García-Sastre A Lu S. Heterologous HA DNA vaccine prime–inactivated influenza vaccine boost is more effective than using DNA or inactivated vaccine alone in eliciting antibody responses against H1 or H3 serotype influenza viruses. Vaccine (2008) 26:3626–33. doi: 10.1016/j.vaccine.2008.04.073 PMC2802517 18538900

[14] van Diepen MT Chapman R Douglass N Galant S Moore PL Margolin E. Prime-boost immunizations with DNA, modified vaccinia virus Ankara, and protein-based vaccines elicit robust HIV-1 tier 2 neutralizing antibodies against the CAP256 superinfecting virus. J Virol (2019) 93:e02155–18. doi: 10.1128/JVI.02155-18 PMC6450106 30760570

[15] Logunov DY Dolzhikova IV Zubkova OV Tukhvatullin AI Shcheblyakov DV Dzharullaeva AS. Safety and immunogenicity of an rAd26 and rAd5 vector-based heterologous prime-boost COVID-19 vaccine in two formulations: two open, non-randomised phase 1/2 studies from Russia. Lancet (2020) 396:887–97. doi: 10.1016/S0140-6736(20)31866-3 PMC7471804 32896291

[16] Sahin U Muik A Vogler I Derhovanessian E Kranz LM Vormehr M. BNT162b2 induces SARS-CoV-2-neutralising antibodies and T cells in humans. Nature (2021) 595(7868):572–577. doi: 10.1038/s41586-021-03653-6 34044428

[17] Woldemeskel BA Garliss CC Blankson JN. SARS-CoV-2 mRNA vaccines induce broad CD4+ T cell responses that recognize SARS-CoV-2 variants and HCoV-NL63. J Clin Invest (2021) 131 :e149335. doi: 10.1172/JCI149335 PMC8121504 33822770

[18] Vaine M Wang S Hackett A Arthos J Lu S. Antibody responses elicited through homologous or heterologous prime-boost DNA and protein vaccinations differ in functional activity and avidity. Vaccine (2010) 28:2999–3007. doi: 10.1016/j.vaccine.2010.02.006 20170767 PMC2847033

[19] Zhang M Zhang L Zhang C Hong K Shao Y Huang Z. DNA Prime-protein boost using subtype consensus env was effective in eliciting neutralizing antibody responses against subtype BC HIV-1 viruses circulating in China. Hum Vaccines Immunother (2012) 8:1630–7. doi: 10.4161/hv.21648 PMC3601137 23111170

[20] Malherbe DC Vang L Mendy J Barnette PT Spencer DA Reed J. Modified adenovirus prime-protein boost clade c HIV vaccine strategy results in reduced viral DNA in blood and tissues following tier 2 SHIV challenge. Front Immunol (2020) 11:626464. doi: 10.3389/fimmu.2020.626464 33658998 PMC7917243

[21] He Q Mao Q An C Zhang J Gao F Bian L. Heterologous prime-boost: breaking the protective immune response bottleneck of COVID-19 vaccine candidates. Emerg Microbes Infect (2021) 10:629–37. doi: 10.1080/22221751.2021.1902245 PMC8009122 33691606

[22] Zhang J He Q An C Mao Q Gao F Bian L. Boosting with heterologous vaccines effectively improves protective immune responses of the inactivated SARS-CoV-2 vaccine. Emerg Microbes Infect (2021) 10:1598–608. doi: 10.1080/22221751.2021.1957401 PMC8381941 34278956

[23] Li J Hou L Guo X Jin P Wu S Zhu J. Heterologous AD5-nCOV plus CoronaVac versus homologous CoronaVac vaccination: a randomized phase 4 trial. Nat Med (2022) 28:401–9. doi: 10.1038/s41591-021-01677-z PMC8863573 35087233

[24] WHO. Status of COVID-19 vaccines within WHO EUL/PQ evaluation process updated.

[25] Schmidt T Klemis V Schub D Mihm J Hielscher F Marx S. Immunogenicity and reactogenicity of heterologous ChAdOx1 nCoV-19/mRNA vaccination. Nat Med (2021) 27:1530–5. doi: 10.1038/s41591-021-01464-w PMC8440177 34312554

[26] Chiu NC Chi H Tu YK Huang YN Tai YL Weng SL. To mix or not to mix? a rapid systematic review of heterologous prime-boost covid-19 vaccination. Expert Rev Vaccines (2021) 20:1211–20. doi: 10.1080/14760584.2021.1971522 PMC8425437 34415818

[27] Barros-Martins J Hammerschmidt SI Cossmann A Odak I Stankov MV Morillas Ramos G. Immune responses against SARS-CoV-2 variants after heterologous and homologous ChAdOx1 nCoV-19/BNT162b2 vaccination. Nat Med (2021) 27:1525–9. doi: 10.1038/s41591-021-01449-9 PMC8440184 34262158

[28] Nie J Li Q Wu J Zhao C Hao H Liu H. Establishment and validation of a pseudovirus neutralization assay for SARS-CoV-2. Emerg Microbes Infect (2020) 9:680–6. doi: 10.1080/22221751.2020.1743767 PMC7144318 32207377

[29] Xu X Nie S Wang Y Long Q Zhu H Zhang X. Dynamics of neutralizing antibody responses to SARS-CoV-2 in patients with COVID-19: an observational study. Signal Transduct Target Ther (2021) 6:197. doi: 10.1038/s41392-021-00611-6 34006847 PMC8129700

[30] Lu S Zhao Y Yu W Yang Y Gao J Wang J. Comparison of nonhuman primates identified the suitable model for COVID-19. Signal Transduct Target Ther (2020) 5:157. doi: 10.1038/s41392-020-00269-6 32814760 PMC7434851

[31] Liu L Wei Q Lin Q Fang J Wang H Kwok H. Anti-spike IgG causes severe acute lung injury by skewing macrophage responses during acute SARS-CoV infection. JCI Insight (2019) 4 :e123158. doi: 10.1172/jci.insight.123158 PMC6478436 30830861

[32] Khoury DS Cromer D Reynaldi A Schlub TE Wheatley AK Juno JA. Neutralizing antibody levels are highly predictive of immune protection from symptomatic SARS-CoV-2 infection. Nat Med (2021) 27:1205–11. doi: 10.1038/s41591-021-01377-8 34002089

[33] McMahan K Yu J Mercado NB Loos C Tostanoski LH Chandrashekar A. Correlates of protection against SARS-CoV-2 in rhesus macaques. Nature (2021) 590:630–4. doi: 10.1038/s41586-020-03041-6 PMC7906955 33276369

[34] He Q Mao Q Peng X He Z Lu S Zhang J. Immunogenicity and protective efficacy of a recombinant protein subunit vaccine and an inactivated vaccine against SARS-CoV-2 variants in non-human primates. Signal Transduct Target Ther (2022) 7:69. doi: 10.1038/s41392-022-00926-y 35241645 PMC8892123

[35] Ewer KJ Barrett JR Belij-Rammerstorfer S Sharpe H Makinson R Morter R. T Cell and antibody responses induced by a single dose of ChAdOx1 nCoV-19 (AZD1222) vaccine in a phase 1/2 clinical trial. Nat Med (2021) 27:270–8. doi: 10.1038/s41591-020-01194-5 33335323

[36] Qi H Chen X Chu C Liu D Ma W Wang Y. Tfh cell differentiation and their function in promoting b-cell responses. Adv Exp Med Biol (2014) 841:153–80. doi: 10.1007/978-94-017-9487-9_6 25261207

[37] Piadel K Haybatollahi A Dalgleish AG Smith PL. Selection and T-cell antigenicity of synthetic long peptides derived from SARS-CoV-2. J Gen Virol (2022) 103 :001698. doi: 10.1099/jgv.0.001698 PMC8895615 35014605

[38] Eickhoff CS Terry FE Peng L Meza KA Sakala IG Van Aartsen D. Highly conserved influenza T cell epitopes induce broadly protective immunity. Vaccine (2019) 37:5371–81. doi: 10.1016/j.vaccine.2019.07.033 PMC6690779 31331771

[39] WHO. WHO/BS.2020.2403 establishment of the WHO international standard and reference panel for anti-SARS-CoV-2 antibody (Accessed November 18).

[40] Zhang M Zhang S. T Cells in fibrosis and fibrotic diseases. Frontiers in Immunology (2020) 11:1142. doi: 10.3389/fimmu.2020.01142 32676074 PMC7333347

[41] Agita A Alsagaff MT. Inflammation, immunity, and hypertension. Acta Med Indonesiana (2017) 49:158–65. doi: 10.1161/HYPERTENSIONAHA.110.163576 28790231

[42] Bian L Gao F Zhang J He Q Mao Q Xu M. Effects of SARS-CoV-2 variants on vaccine efficacy and response strategies. Expert Rev Vaccines (2021) 20:365–73. doi: 10.1080/14760584.2021.1903879 PMC8054487 33851875

[43] Bian L Gao Q Gao F Wang Q He Q Wu X. Impact of the delta variant on vaccine efficacy and response strategies. Expert Rev Vaccines (2021) 20:1201–9. doi: 10.1080/14760584.2021.1976153 PMC8442750 34488546

[44] Wang WB Liang Y Jin YQ Zhang J Su JG Li QM. E484K mutation in SARS-CoV-2 RBD enhances binding affinity with hACE2 but reduces interactions with neutralizing antibodies and nanobodies: Binding free energy calculation studies. J Mol Graphics Model (2021) 109:108035. doi: 10.1016/j.jmgm.2021.108035 PMC8447841 34562851

[45] Costa Clemens SA Weckx L Clemens R Almeida Mendes AV Ramos Souza A Silveira MBV. Heterologous versus homologous COVID-19 booster vaccination in previous recipients of two doses of CoronaVac COVID-19 vaccine in Brazil (RHH-001): a phase 4, non-inferiority, single blind, randomised study. Lancet (2022) 399:521–9. doi: 10.1016/S0140-6736(22)00094-0 PMC8782575 35074136

